# Annotations

**Published:** 1907-10-26

**Authors:** 


### Annotations.

### The Calmette Serum Reaction in Tuberculosis.

In the early part of the present year Professor Calmette published a note on a method for the easy and certain diagnosis of the presence of tubercle in the human body. The method was simplicity itself. It consisted merely in placing on the conjunctiva a drop or two of an aqueous, one per cent. solution of dried tuberculin. If there is no apparent result it may be concluded that the patient is free from tubercle. On the other hand, a local reaction may be considered as proof positive that tubercle is present somewhere in the body. This local reaction appears in the form of a fairly acute muco-purulent conjunctivitis, the beginnings of which may be seen about three hours after the application to the conjunctiva has been made; it usually lasts for some hours, and may be even more prolonged, but at the latest it disappears in the course of a few days and the amount of discomfort suffered by the patient is a mere minimum quantity. All this, it must be admitted, has a very tempting sound, and some may be disposed to ask whether it is not too good to be true. There are so many conditions in which a confident diagnosis of tubercle, even when suspicions are both acute and active, cannot be given, that help in the direction indicated will be of the highest value. The profession has had more than one disappointment in reference to tubercle, and the incredulous may be disposed to regard the present claim with suspicion. Yet it may be remembered that whatever be the therapeutic failure of tuberculin its powers as a diagnostic agent are beyond question, and Calmette's proposal is, after all, a mere modification in the method of application. Of course, the final word must be provided by experience, and as the test is so readily applied this ought soon to be supplied. Moreover, a number of Continental observers support Calmette's claims, and recently Mr. Sydney Stephenson has published a series of ocular cases, in which the new test was applied with apparent success.

### Professor Finlay on Alcohol in Medicine.

In opening the winter session at Aberdeen University, Professor Finlay lectured to the medical students on the position and function of alcohol in the treatment of disease. Referring to a celebrated memorial which appeared some months ago, he said that no valid reason for the issue of this had been advanced, and there might possibly be some truth in the surmise that the origin of the movement could be traced to other than a medical source. As a contrast to this Professor Finlay directed attention to a manifesto issued some twenty-five years ago, and signed by more than 250 of the leading physicians and surgeons of the day. The signatories in this case urged the necessity of regarding alcohol as a powerful drug, to be prescribed only under the sense of a grave responsibility and with the details and precautions which such a view of its nature suggested. They also expressed the opinion that many people immensely exaggerate the value of alcohol as an article of diet, and that medical men ought to use their influence to inculcate habits of great moderation in the use of alcoholic liquors. Professor Finlay expressed his own general agreement with this view, and stated that as the years pass he has come to use alcohol less and less in the treatment of disease. While firmly believing that occasions exist in which the value of alcohol as a drug is undoubted, he is even more strongly convinced that the common and routine prescription of it in all acute cases is not only unnecessary but harmful, whilst in chronic forms of disease there is little or no need for it. In young patients, even in acute illnesses, Professor Finlay finds alcohol to be rarely necessary, and even in those of riper years many are better without it. The "high stimulation" practice, which was particularly associated with the name of Dr. Todd, of King's College Hospital, has long since disappeared, and the movement and tendency to decrease or limit still further the use of alcohol in disease is still in progress. Professor Finlay paid an eloquent tribute to the work of the late Sir Wm. Gairdner in this direction. It was the reasonable and forcible arguments of this great physician, founded on a vast experience of typhus fever, which compelled the profession to modify its routine use of alcohol in all cases of febrile disease.

### What is Special Practice?

In a series of new rules proposed for a large provincial hospital it is demanded that the physicians and surgeons who occupy positions on the staff in connection with special forms of practice shall confine themselves strictly to their several departments. A proposal of a similar nature has also received favourable consideration from others concerned with the organisation and management of hospitals. This suggestion on the face of it sounds reasonable enough, yet we will venture to say it would be found very difficult to apply in actual work. To the layman it appears quite simple to classify cases as examples, say, of eye, throat, ear, and skin diseases, and to send the patient in each instance to the appropriate department. But is it so in practice? We think not, and the matter can be readily tested by a visit to the special hospitals or special departments of the general hospitals in the metropolis. Probably for this purpose the former are to be preferred; certainly there are to be found in the out-patient departments of these institutions patients who only by a very peculiar interpretation of the term can be regarded as the victims of local disease. Is, for example, an optic nerve atrophy or an ocular paralysis an "ophthalmic" case in the sense that it is suitable for treatment by an ophthalmic surgeon? or ought such cases to be referred to the neurologist or to a general physician? Take, again, a laryngeal paralysis or a case of nerve deafness, and a similar difficulty presents itself. And is it not true that cases of Graves' disease may be found sometimes at an ophthalmic hospital, sometimes in the throat department, and sometimes in charge of the physician? These facts, and many more might be quoted, are sufficient to show how difficult it is to divide practice arbitrarily into departments corresponding with the popular specialities of the day.

